# Intracranial pressure following surgery of an unruptured intracranial aneurysm—a model for normal intracranial pressure in humans

**DOI:** 10.1186/s12987-024-00549-1

**Published:** 2024-05-21

**Authors:** Nicolas Hernandez Norager, Alexander Lilja-Cyron, Casper Schwartz Riedel, Anders Vedel Holst, Sarah Hornshoej Pedersen, Marianne Juhler

**Affiliations:** 1 Clinic of Neurosurgery, Copenhagen University Hospital, Inge Lehmanns Vej 6, 2100 Copenhagen East, Denmark; 2 Clinic of Neurosurgery, Aarhus University Hospital, Aarhus, Denmark

**Keywords:** Telemetric ICP monitoring, Intracranial pressure (ICP), Unruptured intracranial aneurysm, Normal ICP reference value

## Abstract

**Objective:**

Optimizing the treatment of several neurosurgical and neurological disorders relies on knowledge of the intracranial pressure (ICP). However, exploration of normal ICP and intracranial pressure pulse wave amplitude (PWA) values in healthy individuals poses ethical challenges, and thus the current documentation remains scarce. This study explores ICP and PWA values for healthy adults without intracranial pathology expected to influence ICP.

**Methods:**

Adult patients (age > 18 years) undergoing surgery for an unruptured intracranial aneurysm without any other neurological co-morbidities were included. Patients had a telemetric ICP sensor inserted, and ICP was measured in four different positions: supine, lateral recumbent, standing upright, and 45-degree sitting, at day 1, 14, 30, and 90 following the surgery.

**Results:**

ICP in each position did not change with time after surgery. Median ICP was 6.7 mmHg and median PWA 2.1 mmHg in the supine position, while in the upright standing position median ICP was − 3.4 mmHg and median PWA was 1.9 mmHg. After standardization of the measurements from the transducer site to the external acoustic meatus, the median ICP_midbrain_ was 8.3 mmHg in the supine position and 1.2 mmHg in the upright standing position.

**Conclusion:**

Our study provides insights into normal ICP dynamics in healthy adults following a uncomplicated surgery for an unruptured aneurysm. These results suggest a slightly wider normal reference range for invasive intracranial pressure than previously suggested, and present the first normal values for PWA in different positions. Further studies are, however, essential to enhance our understanding of normal ICP.

*Trial registration* The study was preregistered at www.clinicaltrials.gov (NCT03594136) (11 July 2018)

**Supplementary Information:**

The online version contains supplementary material available at 10.1186/s12987-024-00549-1.

## Introduction

Intracranial pressure (ICP) plays a vital role both in normal life, and in a spectrum of acute neurosurgical and neurological disorders, such as intracranial haemorrhages and traumatic brain injury, as well as more chronic conditions, such as normal pressure hydrocephalus and idiopathic intracranial hypertension [1–4]. Invasive measurement of ICP is routinely used in managing these patients, and ICP-lowering treatment is used to reach an acceptable ICP. Despite this, the current literature on normal ICP values remains scarce. Thus, in a recent systematic review by our department, we pooled the available literature and found insufficient data to establish a clinically applicable reference range for normal ICP [5].

Pursuing a normal range of ICP poses a dual challenge. Firstly, the ethical restraints arising from the invasive nature of ICP measurement hinder obtaining values from healthy individuals [6]. Secondly, the invasive procedure of implanting an ICP measurement device can potentially influence the ICP itself [7]. Previous studies involving ‘pseudo-healthy’ subjects have primarily been based on two groups of patients. (1) Patients who underwent ICP measurement but did not subsequently receive an ICP-related diagnosis. Thus, these patients had symptoms indicating increased ICP but no apparent ICP disturbance upon measuring ICP [8–10]. (2) Healthy patients with measurement of the lumbar cerebrospinal fluid (CSF) pressure [11–13]. However, the feasibility of lumbar CSF pressure as a surrogate for ICP remains a subject of ongoing debate, especially in patients with obstructive lesions. Additionally, it has been shown that the lateral recumbent body position during the procedure may cause elevated ICP [14–16]. Devices for telemetric ICP monitoring facilitate repeated non-invasive ICP measurements months after implantation of the device, thus allowing investigation of ICP in new patient populations [17, 18]. In a study published in 2014, Andresen et al. inserted telemetric ICP sensors in patients who had a small, demarcated brain tumors removed. The rationale of the study was to measure ICP repeatedly in the weeks after surgery when there were no longer structural abnormalities influencing ICP and when the possible impact from surgery on ICP was expected to have passed [19]. Finding a median ICP of 0.5 mmHg in supine position and − 3.7 mmHg in upright standing position they challenge the previously accepted normal reference range [5, 13]. If these values hold for the general population, treatment targets for various disorders might need reevaluation. However, confirming these findings mandates broader and more heterogenic study cohorts.

Understanding the nuances of ICP in healthy individuals is crucial; and thus our study aims to explore normal ICP dynamics via telemetric ICP monitoring in patients undergoing surgery for an unruptured intracranial aneurysm.

## Methods

### Study design and population

We conducted a prospective observational study including adult patients (age > 18 years) undergoing surgery for an unruptured intracranial aneurysm. The neurosurgical intervention when clipping an unruptured aneurysm without complications is thought to have no long-term influence on ICP and CSF dynamics. This patient group is unique as the patients are neurologically stable and yet require an invasive neurosurgical procedure, providing an ideal context to measure 'normal' intracranial pressure unaffected by acute brain injury or altered neurological status. Following successful and uncomplicated surgery, the patient is thus considered pseudo-healthy without intracranial pathology. Strict inclusion and exclusion criteria were implemented to minimize potential confounding factors affecting ICP (Table 1).

**Table 1:** Inclusion and exclusion criteria

Inclusion criteria
1. Requiring surgery for an unruptured cerebral aneurysm
2. Age 18 to 80 year

Exclusion criteria
1. Previous head trauma or intracranial hemorrhage
2. Current or previous examination or treatment for hydrocephalus
3. Current or previous examination or treatment for idiopathic intracranial hypertension
4. Previous operation with insertion of cerebrospinal fluid shunt
5. Global cerebral edema
6. Increased infection risks
7. Surgical complications during the primary operation
8. Unable to understand patient information

After the commencement of our study, we encountered issues related to sensor drift. These problems manifested after the inclusion of our patient cohort, and we did not observe significant drift within our sensors. Two sensors suspected of drift were tested post-explanation using the method described in Norager et al., but no meaningful drift was found (< 1 mmHg) [20].

### Surgical intervention

**Fig. 1:**
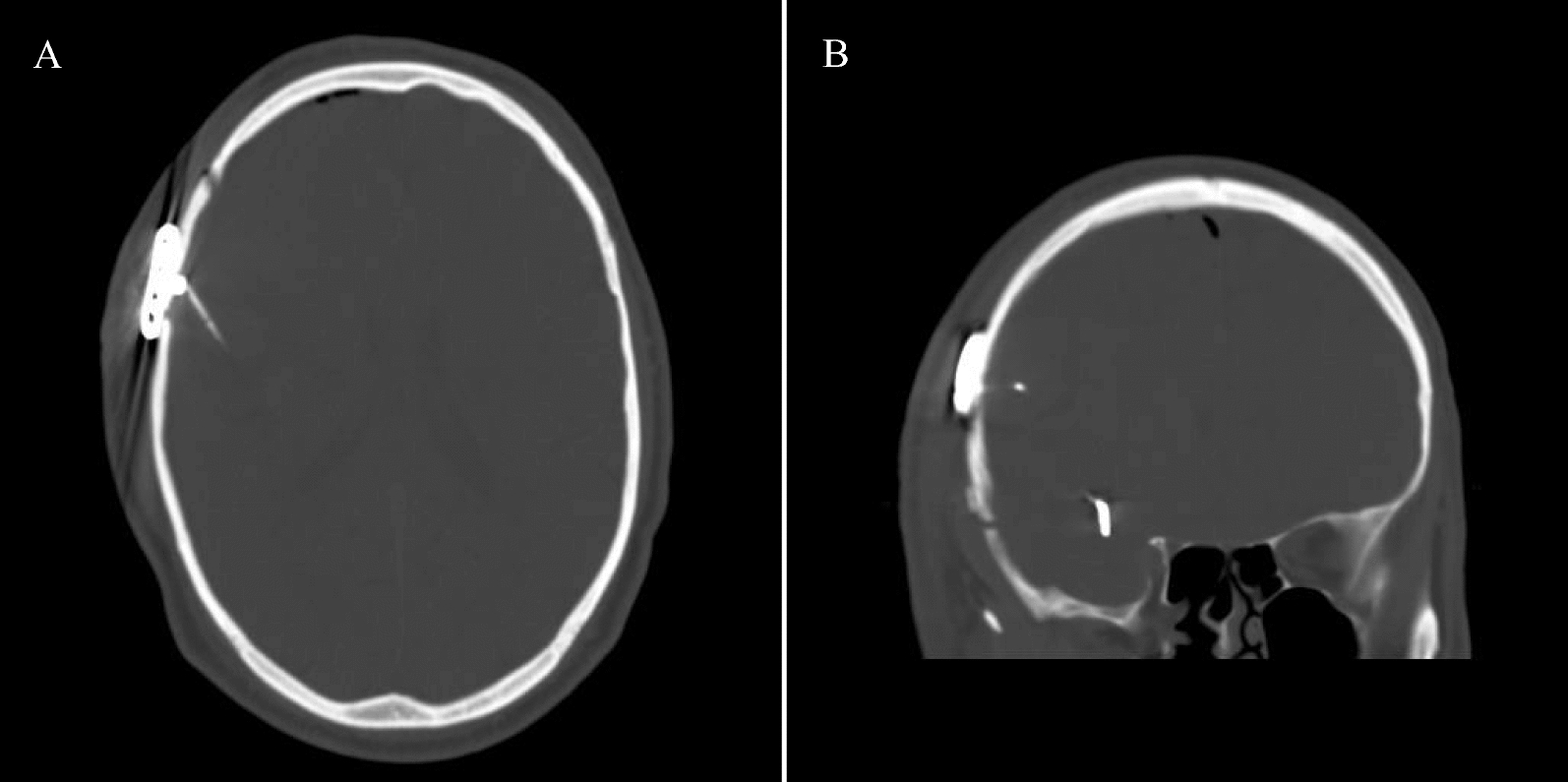
CT scan of telemetric ICP sensor. The figure displays coronal and axial CT scans of a patient, highlighting the implanted telemetric sensor. Routine postoperative CT scans following the clipping of unruptured aneurysms are not standard practice within our neurosurgical department and are reserved for cases where complications are suspected. Consequently, the CT scan depicted in this figure originates from a patient who was excluded due to postoperative bleeding

A telemetric ICP sensor (Neurovent-P-tel, RAUMEDIC AG, Germany, Helmbrechts) was implanted subsequently to surgical clipping of the unruptured aneurysm, given that no complications arose during the aneurysm surgery. The ICP sensor was inserted 2 cm into the brain parenchyma through a cranial burr hole at the edge of the craniotomy (see Fig. 1). Removal of the ICP sensor was performed in 6/6 cases. One case necessitated early removal on day 30 post-implantation due to the development of a cutaneous defect. The remainder were explanted after end follow-up, unrelated to complications.

### Data collection

Following implantation, ICP was measured on day 1, day 14, day 30, and day 90, thus facilitating long-term evaluation of ICP fluctuations and development following the surgical intervention. On day 1, we tested the ICP sensor, but did not perform long-term measurements due to pain from the surgical wound when applying the percutaneous reader unit. On day 14, day 30, and day 90, a standardized postural measurement protocol was conducted, including four body positions (supine, lateral recumbent with the neck extended, standing upright, and 45-degree sitting). The lateral recumbent position was conducted with the patient lying either on the contralateral side of the implanted ICP sensor. Sampling of ICP was started once the signal had stabilized after transitioning to a new position (approximately 5 min) [21]. ICP was measured for 10 min in each position to further accommodate potential transient ICP disturbances due to the changes in body position. Artifacts in the ICP recording caused by physical events, such as coughing and changes in neck position, were annotated for later removal in the data analysis.

### Statistical analysis

All statistical analyses were performed using RStudio (R 3.4.1, R Development Core Team [2008], Vienna, Austria). Prior to commencing the study, we calculated the required sample size using the methodology described by Hulley et al. [22], and found that a cohort of a minimum of 20 patients was required to reliably determine whether the current used reference values were accurate with 80% power and an alpha level of 0.05. However, challenges in obtaining informed consent, compounded by the issues with sensor drift, extended the study duration and ultimately prevented us from reaching the planned number of patients. Given the limited number of patients, our statistical evaluation was confined to descriptive statistics. We present the median ICP for each position along with the interquartile range (IQR) and ICP standardized to the external acoustic meatus, referred to as ICP_midbrain_ (see supplementary material). Furthermore, we calculated intracranial pressure pulse wave amplitude (PWA) for each position. PWA was calculated as described in Norager et al. [23].

### Ethics

Patients were informed prior to the scheduled procedure, and a written consent was obtained before inclusion. The study adhered to the Helsinki Declaration (1964) and was approved by the Scientific Ethics Committee of the Capital Region in Denmark (H-17011472) and the Danish Data Protection Agency. The study was preregistered at www.clinicaltrials.gov before inclusion of the first patient (NCT03594136) (11 July 2018).

## Results

### Patients

Six patients had an ICP sensor inserted. All sensors functioned when tested on day 1 post-implantation. However, one patient was excluded due to postsurgical bleeding and thus not regarded as healthy in terms of ICP dynamics. Another encountered skin erosion approximately 1 month post-surgery, preventing the collection of the 90-day measurement. Of the included patients, 3/5 were female (all postmenopausal), the mean age was 62 years, and the mean BMI was 23. Detailed demographic information and details regarding the clipped aneurisms is available in Table 2.

**Table 2:** Demographics

	Gender	Age	BMI	Previous neurological	Comorbidities	Medications	Aneurysm (location and size)
Patient 1	F	54	23	None	None	None	MCA^a^ bifurcation, 5 mm
Patient 2	F	55	21	None	DM1	Insulin	Top ICA^b^, 10 mm
Patient 3	F	56	23	Epilepsy	Hypertension, epilepsy, hyperthyroidism	Enalabril, levetiracetam, centyl, thiamazol	ACOM^c^, 7 mm
Patient 4	M	74	27	None	Hypertension, ischemic heart disease	Atorvastatin, amlodipin, omeprazol	ACOM, 5 mm
Patient 5	M	69	22	None	Hypertension, hypercholesterol	Losartan, atorvastatin	MCA bifurcation, 9 mm

^a^Middle cerebral artery

^b^Internal carotid artery

^c^Anterior communicating artery

### Intracranial pressure

**Fig. 2:**
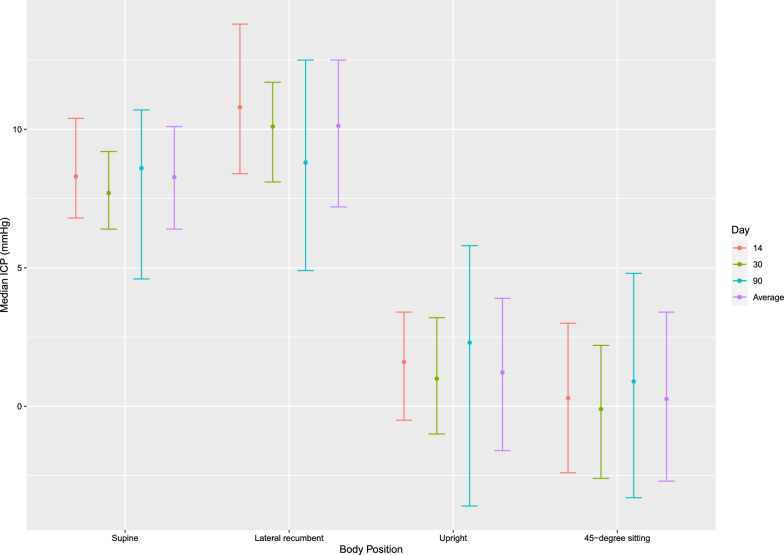
Median intracranial pressure in different body positions. The figure shows the median ICP values zeroed to the external acoustic meatus with corresponding 25th and 75th quartile ranges for each monitoring day (e.g., days 14, 30, and 90). These values are further subdivided by body positions: supine, lateral recumbent, standing upright, and 45-degree sitting. The median ICP for each body position across monitoring days is also presented

**Fig. 3:**
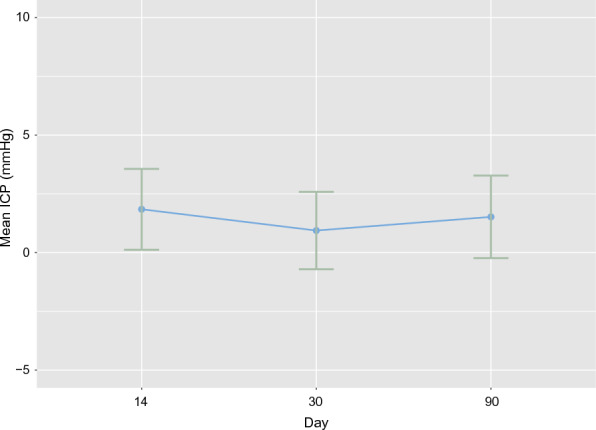
Mean intracranial pressure across monitoring days. The figure displays the aggregated mean ICP, compiled from all patients and body positions, for each day of monitoring. Mean ICP with coherent 95% confidence intervals across various monitoring days are displayed. No meaningful difference in ICP across monitoring days is found

Tables 3 and 4 provides a comprehensive presentation of median ICP values measured across different body positions. Notably, measured median ICP were negative in 45 degrees sitting (− 2.9 mmHg, IQR 6.1) and standing position (− 3.4 mmHg, IQR 5.5), contrasting with positive values in supine (6.7 mmHg, IQR 3.7) and lateral recumbent position (8.0 mmHg, IQR 5.3). Upon correction of zero point to the external acoustic meatus, median ICP_midbrain_ was positive in both 45 degrees sitting (0.3 mmHg, IQR 6.1) and standing (1.2 mmHg, IQR 5.5), as well as supine (8.3 mmHg, IQR 3.7) and lateral recumbent position (10.1 mmHg, IQR 5.3). Changing body position from supine to standing, we observed a decrease in ICP of 10.1 mmHg (see Fig. 2). Analyzing longitudinal ICP data across monitoring days from all patients and body positions, we found a stable ICP, with a mean ICP at day 14 of 1.6 mmHg (IQR 3.5), day 30 of 1.3 mmHg (IQR 3.6), and day 90 of 1.8 mmHg (IQR 3.3) (see Fig. 3).

**Table 3:** Median intracranial pressure subdivided into monitoring time and body position

	No. of patients	Supine ICP	Lateral recumbent ICP	Standing ICP	45-degree sitting ICP
Day 14	5	5.7 (4.2–7.8)	8.7 (6.3–11.7)	− 3.0 (− 5.1 to − 1.2)	− 3.9 (− 6.6 to − 1.2)
Day 30	5	5.1 (3.8–6.6)	8.0 (6.0–9.6)	− 3.6 (− 5.6 to − 1.4)	− 4.3 (− 6.8 to − 2.0)
Day 90	4	6.0 (2.0–8.1)	6.7 (2.8–10.4)	− 2.3 (− 8.2 to 1.2)	− 3.3 (− 7.5 to 0.6)

All values are presented in mmHg and as median (interquartile range)

**Table 4:** Median intracranial pressure, intracranial pressure zeroed to the external acoustic metus and intracranial pressure pulse wave amplitude in different body position

	Supine	Lateral recumbent	Standing	45-degree sitting
Median ICP^a^	5.7 (3.8–7.5)	8.0 (5.1–10.4)	− 3.4 (− 6.2 to − 0.7)	− 3.9 (− 6.9 to − 0.9)
Median ICP_midbrain_	8.3 (6.4–10.1)	10.1 (7.2–12.5)	1.2 (− 1.6 to 3.9)	0.3 (− 2.7 to 3.4)
Median PWA^b^	2.1 (1.5–2.7)	2.3 (1.4–3.2)	1.9 (1.2–2.6)	1.8 (1.3–2.3)

All values are presented in mmHg and as median (25th–75th quartile)

^a^Intracranial pressure

^b^Intracranial pressure pulse wave amplitude

### Pulse wave amplitude

**Fig. 4:**
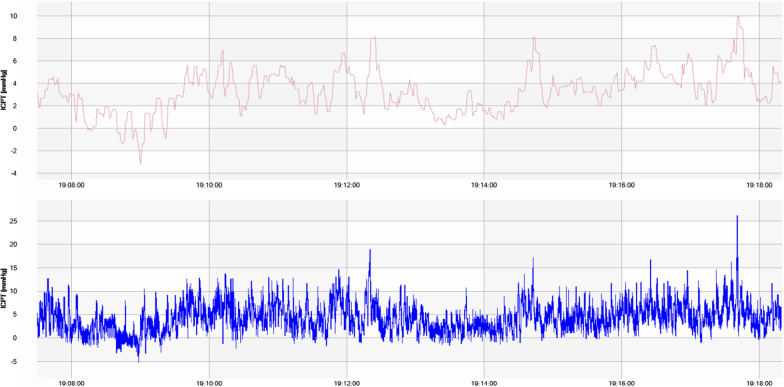
Ten-minute segment of ICP measurement. The figure presents a ten-minute ICP recordings obtained via the telemetric ICP sensor. The sensor samples ICP data at a sampling rate of 5 Hz, which is sufficient for monitoring fluctuations in pulse wave amplitude. However, this frequency is suboptimal for conducting detailed analysis of the pulse wave morphology

In Table 4, median PWA in different body positions are presented. We see only small changes in median PWA across body position with the lowest median PWA in 45 degree sitting position of 1.8 mmHg (IQR 1.0), and the highest PWA in lateral recumbent position of 2.3 mmHg (IQR 1.8). In supine, we found a median PWA of 2.1 mmHg (IQR 1.2) and in standing position a median PWA of 1.9 mmHg (IQR 1.4). In Fig. 4, we present an illustrative 10-min segment of ICP measurement, wherein the PWA is visually depicted.

## Discussion

This study reports parenchymal ICP and PWA values from telemetric ICP measurements in five patients who underwent uncomplicated surgery for an unruptured intracranial aneurysm. This setting is as close to invasive ICP measurement in normal individuals as ethically acceptable. Our findings are consistent with a similar study conducted by our study group, stating that normal ICP values are lower than traditionally accepted and that parenchymal ICP is slightly negative in the standing upright position [19].

Upon calibration to the level of the external acoustic meatus, we report a median supine ICP_midbrain_ of 8.3 mmHg and a median standing upright ICP_midbrain_ of 1.2 mmHg. This is in accordance with the recently established reference values from a systematic review by our team [5]. The systematic review reported a mean supine ICP of 8.6 mmHg (reference interval: 0.9–16.3 mmHg) and a mean upright ICP of 1.0 mmHg (reference interval − 5.9 to 8.3 mmHg), when only including the studies with intracranially obtained measurements, thus challenging the conventionally used reference range of 7 to 15 mmHg [13]. The conventional interval relies mainly on lumbar CSF pressure being used as a proxy for ICP or extrapolations from patients with presumed ICP abnormalities prior to monitoring but no apparent ICP disturbance upon measuring ICP [8, 13, 24, 25]. Therefore, whether these values in fact represent normal physiology remains questionable. Furthermore, we see that our reference interval is in fact broader than the previously described. This might be attributed to the limited data available and the small cohort size, which inherently leads to statistical variability. Furthermore, due to the current scarcity of data material on normal ICP it might be that variance of ICP in healthy individuals is in fact broader than previously thought. Further research with larger sample sizes is necessary to refine the reference range for ICP.

To our knowledge, this is the first study to provide normal values for PWA in different body positions. We found a median PWA of 2.1 mmHg in the supine position and a mean PWA of 1.9 mmHg in the standing upright position. The relative stability of PWA, irrespective of relative large changes of ICP, diverges from the classical expectation of PWA increasing proportionally to ICP [26, 27]. However, previous studies have predominantly examined the ICP-PWA relationsship in supine position [28]. A single study examined the changes in PWA during positional shift, and similarly found minimal changes in PWA during positional changes [23].

A probable reason for these relatively small changes in PWA can be found if we further examine the constituents of PWA. In essence, PWA represents the pressure changes within the intracranial compartment stemming from the volume influx caused by the cardiac pulse. Since our cohort was presumably healthy in terms of ICP, they were on the compliant far left side of the pressure–volume curve, and thus had high intracranial compliance. According to the pressure–volume curve, in this instance moderate changes in intracranial volume do not translate into significant pressure changes, consequently the short volume increase representing the PWA only results in slight changes in pressure. Furthermore, we have to address the physiological changes occurring when changing from supine to standing position. This transition impacts the cardiovascular system by reducing venous return and ventricular stroke volume, altering baroreceptor pressure, activating extracranial baroreceptor reflexes, increasing heart rate via reduced vagal tone, increasing peripheral vascular resistance, and adjusting cerebral perfusion mechanisms. These changes collectively elevate the heart rate and blood pressure, reduce stroke volume, and either stabilize or slightly reduce cardiac output while maintaining cerebral blood flow through compensatory mechanisms [29]. Thus, the intracranial volume influx from each cardiac pulse is lowered, thus dampening the amplitude of the PWA. Interesting, the correlation between ICP and PWA in standing position has been investigated, and a linear correlation has been established [23]. In conclusion, changes in ICP in a given position leads to an increase in PWA, while the reduction in ICP due to positional shift from supine to standing only leads to minimal reduction in PWA [23].

### Effect of positional changes on ICP

The relationship between ICP and body position has been extensively reported in patients with ICP disorders, with evidence showing a lower ICP in upright position compared to supine [23, 30, 31]. The underlying mechanism likely is the gravitational force inducing a caudal shift of blood and CSF [32, 33]. A previous study quantified this reduction closer, with findings showing a decrease in ICP proportional to the hydrostatic gradient up to a 20-degree head elevation. Beyond this angle, the impact of further head elevation on ICP was only marginal [30, 33]. Notably, the 20-degree elevation coincides with the angle at which the internal jugular vein, which accounts for the main intracranial venous drainage from the brain, collapses [33, 34]. Although our study was not designed to investigate these exact dynamics, our data align with these findings, showing no meaningful difference in ICP between the standing upright and 45-degree sitting positions.

Given that humans spend a considerable amount of time in an upright position, establishing a reference interval for ICP specific to this position is essential for the accurate interpretation of long-term diagnostic monitoring and tailoring treatment. Despite the limited patient population, our study suggest that negative ICP readings may be considered within normal physiological limits which could prove to be utmost important in future shunt designs. Additionally, we found a mean ICP difference of 7.1 mmHg between the supine and standing upright positions after calibrating to the external acoustic meatus. This finding is consistent with previous studies on the matter [33, 35, 36]. The literature regarding ICP in standing position is scarce; however, other existing studies have indicated that negative ICP values may be within the normal range for healthy adults [19, 33, 36, 37]. The negative ICP values does not signify a vacuum but rather a pressure lower than atmospheric pressure. The intracranial compartment is in healthy adults insulated from atmospheric pressure by the cranium. However, following decompressive craniectomy indentation of the skin overlying the removed part of the cranium (s.c. “syndrome of the trephined”) is sometimes seen [38]. This additionally suggests an intracranial pressure lower than atmospheric.

In the context of managing overdrainage in patients with ventriculoperitoneal or ventriculoatrial shunts, recognizing that negative ICP values in the standing position may represent a normal physiological state is of clinical significance. Overdrainage poses diagnostic challenges, partly because established ICP benchmarks for the standing position—or a range of ICP change from supine to standing that would signal overdrainage—have not been well-documented. Within our cohort, transitions from supine to standing did not result in ICP alterations exceeding 9 mmHg. Moreover, our data suggests that negative ICP values—ranging down to − 1.2 mmHg when zeroed to the external acoustic meatus and down to − 6.2 mmHg when parenchymally measured without zeroing—is within normal physiological limits.

Similarly, we found a slight elevation in ICP of approximately 2 mmHg in the lateral recumbent position compared to the supine position. This is comparable to reported in previous literature where the neck was kept straight during lateral recumbent position as in our study. Notably, two recent investigations found a elevation of 7–10 mmHg if the neck was flexed [16, 36]. One of the studies specifically examined flexion of the neck during lateral recumbent position and found this to be the predominant factor behind the elevation in ICP [16]. The flexion of the neck may lead to compression of the internal jugular veins, thus hindering venous outflow from the brain [39, 40]. This gives rise to an interesting discussion about the correlation between lumbar cerebrospinal fluid opening pressure (LCSFop) and intracranially obtained ICP. Various studies have demonstrated excellent correlations between ICP and LCSFop, while others report significant discrepancies [41, 42]. In our study group's previously mentioned systematic review, which examined reference values for ICP and LCSFop, a marginal difference was observed. Across all studies of normal ICP/LCSFop, the cohort measured for LCSFop showed a notably higher pressure by an average of 2.1 mmHg. However, many studies included in this review failed to specify the patients' neck positions during measurements. Given that neck flexion during measurement can influence ICP, this could potentially account for the observed differences [16, 36]. It is therefore plausible that ICP and LCSFop readings are comparable when taken in the same body position and referenced to the same anatomical point, especially in patients without an obstruction in the CSF pathways.

### Reference point of ICP measurement

Our study also revealed a considerable variation in ICP depending solely on the reference point. The selection of a reference point is essential in ICP measurements, as the measurements can be obtained from different locations within the brain. Therefore, in order to facilitate comparisons of ICP measurements, it is necessary to designate a specific location in the brain as the reference point. This essentially means that the measured ICP is adjusted to what it would be if measured at a given reference point. Whereas ventricular ICP measurements are typically standardized to the external acoustic meatus, a may arise with parenchymal ICP sensors. These sensors are, unlike ventricular obtained measurements, not typically standardized to a specific reference point. Instead, they directly measure ICP at the position at the sensor’s tip [43, 44]. This inconsistency may lead to discrepancies in ICP readings, which we found could diverge up to 4.6 mmHg in the standing upright position. In the context of managing acutely elevated ICP, such as in cases of intracranial haemorrhage, the implications of these findings are moderated by most patients being positioned with a neuroprotective 30-degree head elevation rather than in an upright position. For our study, the ICP difference due to reference point zeroing would amount to approximately 2.3 mmHg, which should be considered in clinical settings. However, this would hardly have any meaningful clinical implications, and treatment decisions in these cases also heavily rely on subjective symptoms, clinical presentation, and imaging findings. While cases involving acute substantial ICP elevations demand intervention, the scenario is more intricate when patients exhibit chronically slightly or moderately increased ICP—such as in cases with normal pressure hydrocephalus or the context of refining treatment for shunt patients. In these scenarios, the in-depth comprehension of the basic physiology of ICP can affect treatment modalities and even diagnostics.

### Effect of eliminating positional changes in ICP

As previously states, ICP differs significantly significantly upon different body position, and this discrepancy is probably mostly due to the hydrostatic pressure gradient caused by the gravitational pull. Notably, astronauts in whom gravity and thus the daily ICP variations of positional shifts are eliminated, frequently manifest symptoms associated with increased ICP, such as papilledema and headache [45, 46]. However, a study by Lawley et al. found that the absence of gravity does not increase supine ICP to pathological levels [32]. This paradox suggests that the removal of the gravitational influences on ICP results in the presentation of elevated ICP symptoms, even when single ICP values remain within the normal range. The reason could be that a singular ICP measurement does not encompass the entire clinical context. Instead, the duration spent within specific ICP thresholds may be more critical for understanding the pathophysiology involved in astronauts and maybe other patients.

Furthermore, we hypothesize that a subset of patients, even in the presence of gravitational forces, may exhibit limited venous outflow through the internal jugular vein in an upright position, leading to a diminished variance in ICP with positional changes. These individuals may present with less risk of overdrainage if shunted or with symptoms indicative of elevated ICP, despite only minimal increases in measured ICP. This reflection underscores the necessity for a more nuanced comprehension of ICP dynamics across various body positions, emphasizing the complexity of ICP regulation and its implications for pathological states.

## Limitations

Our study is not without limitations that warrant careful consideration. These limitations mainly include sample size, patient cohort selection, and the use of telemetric ICP sensors. Foremost among these is the limitation imposed by our patient inclusion. Despite the intent to include a sample of 20 patients, the study ultimately consisted of only five patients. As a result, our analysis primarily leans on descriptive statistics, and a comprehensive reference interval could not be established. The small size of our patient cohort hindered the investigation of associations between ICP and parameters such as BMI, blood pressure, and sex. The modest inclusion rate of approximately 10% was the primary reason for our diminished sample size. The limited rate was likely attributed to several factors, most notably patient concerns regarding ICP sensor insertion and the potential risks associated with subsequent removal. Additionally, many patients experienced anxiety related to the primary surgery for the intracranial aneurism and were not interested in further concerns.

Another critical aspect of our study is the specific patient cohort we chose to include. For ethical reasons, we opted for patients needing neurosurgical intervention and included a patient group where the surgical intervention was unlikely to disturb ICP significantly. The investigation of normal ICP introduces the issue of needing to subject patients to neurosurgical interventions before measurement, which runs the risk of changing ICP [7]. While non-invasive ICP monitoring techniques are being developed, their accuracy in depicting accurate ICP remains a work in progress [43, 47]. However, the technological advance in telemetric ICP monitoring enables the investigation of previously unexplored patient cohorts. While telemetric parenchymal ICP monitoring offers distinct advantages for exploring ICP, it should be used with consideration for its limitations, particularly the possibility of ICP sensor drift. We have previously investigated the ICP sensors’ drift and found a median drift of 2.5 mmHg after 241 days, with a clear trend toward more significant drift after a longer implantation time and a substantial drift in sensors placed within a burr hole were a sensor had previously been inserted [48]. However, in our study, we did not reimplant sensors. Furthermore, we did not collect data beyond 90 days after implantation, which is the approved usage period for the telemetric ICP sensor. This limited timeframe diminishes the likelihood of drift playing a substantial role in our data.

## Conclusions

With this study, we explore normal ICP dynamics in pseudo-healthy adults without intracranial pathology and illustrate the importance of a reference point in the accuracy in ICP monitoring. Out limited dataset aligns with previous established reference values and elaboration on postural ICP changes. While unable to establish definitive reference values, our findings contribute with valuable data and doubles existing data on normal ICP and PWA in pseudo-healthy adults. Further investigations with larger cohorts are essential for a comprehensive understanding of normal ICP and PWA, and we suggest the following cohorts: small epilepsy resections, small arteriovenous malformations, or single cavernous angiomas.

### Supplementary Information

Supplementary Material 1.

## Data Availability

Anonymized data are available upon reasonable request from the corresponding author and after clearance by the competent ethics committee.
