# Supplementary material for: Intracranial pressure following surgery of an unruptured intracranial aneurysm—a model for normal intracranial pressure in humans
Source: Fluids Barriers CNS. 2024 May 21;21:44. doi: 10.1186/s12987-024-00549-1 (PMC11110356; doi:10.1186/s12987-024-00549-1)
Supplement: Supplementary file 1 — Supplementary Material 1. [file 12987_2024_549_MOESM1_ESM.docx]

**Standardization of intracranial pressure to the external acoustic meatus**

A correction for the hydrostatic pressure gradient between the sensor's location and the external acoustic meatus is necessary to adjust the reference point of intracranial pressure to the external acoustic meatus. Initially, the estimated sensor positions were inserted into a CT cerebrum scan of the patient, thus allowing us to find the vertical and sagittal dimensions of the hydrostatic column. The hydrostatic pressure difference relies on the patient's position; in a 90-degree upright position, it is solely contingent on the vertical axis, whereas in a 0-degree supine position, it is solely dictated by the sagittal axis. In all positions in between, it is a function of both axes. Mathematically, this relationship is expressed through the sine of the angle of head elevation, e.g., for a 30-degree head tilt, the percentage of the vertical axis' contribution can be expressed as sin(30), whereas the sagittal axis' contribution is represented by sin(60). The assumed density of the brain parenchyma and the standard acceleration of gravity are also needed to calculate the pressure of the hydrostatic column. Thus, the difference in pressure attributable to the hydrostatic column in vertical and sagittal plan (HC_v_ and HC_s_ ) was calculated by:

$$HCv =\left( d*g*h*\sin\left( \alpha\right) \right)*k$$

$$HCs =\left( d*g*h*\sin\left( \beta\right) \right)*k$$

Where:

*HC_v_* = Pressure difference due to the vertical hydrostatic column

HC_s_ = Pressure difference due to the sagittal hydrostatic column

*d* = Density of brain parenchyma (1041.2 kg/m³)

*g* = Standard acceleration due to gravity (9.83 m/s²)

*h* = Height of the hydrostatic column

*α* = Angle of head elevation in the vertical plane

*β* = Angle of head elevation in the sagittal plane

*k* = Conversion constant from Pascal to mmHg (0.0075)

Afterward, we could use the derived hydrostatic pressure gradient to adjust the measured ICP to the external acoustic meatus:

$${ICP}_{midbrain} ={ICP}_{m}+{HC}_{v}+ {HC}_{s}$$

ICP_midbrain_ = ICP referenced to the external acoustic meatus.

ICP_m_ = Measured ICP

Below is a figure illustrating the CT scan process used to determine the external acoustic meatus and measure the hydrostatic column's height in both sagittal and vertical planes.


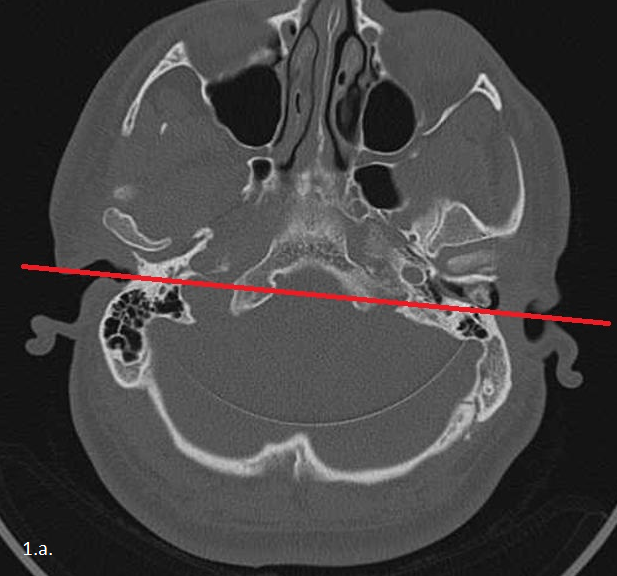

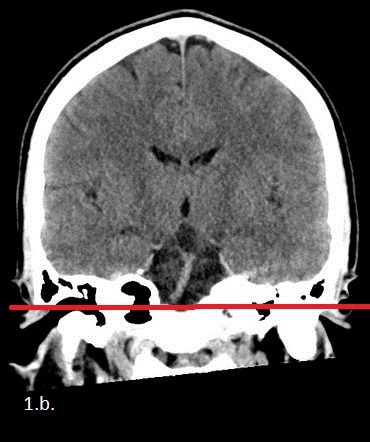


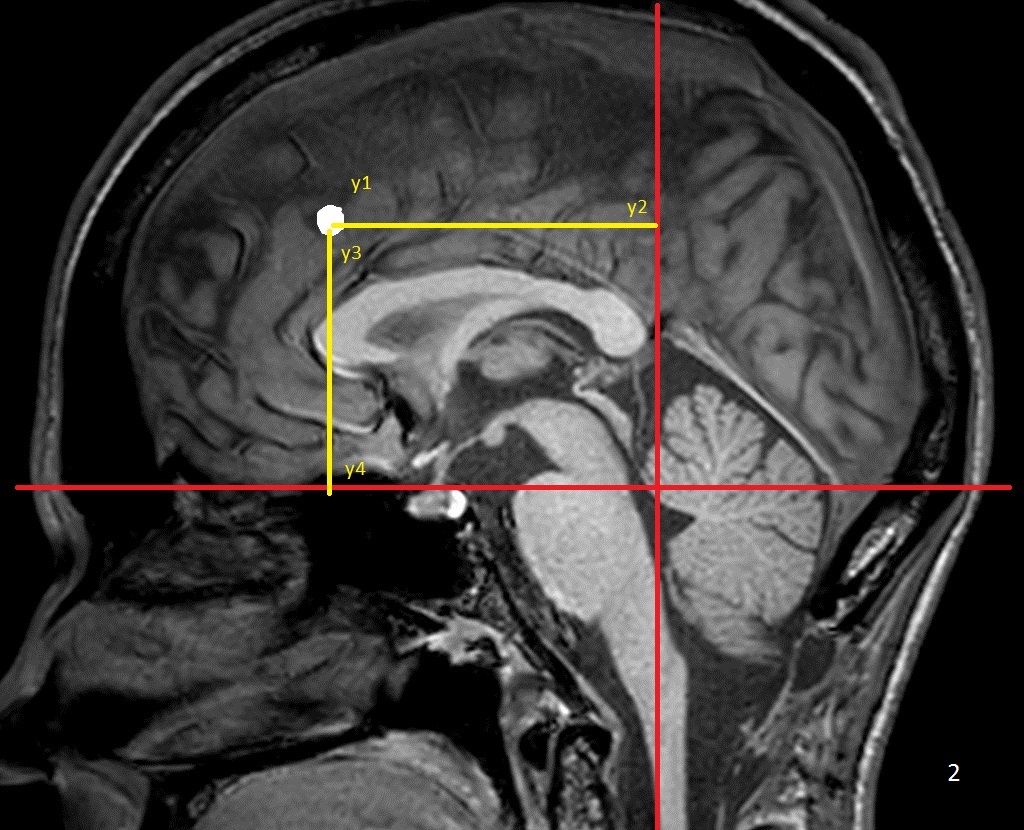


**Figure legend**

Initially, the external acoustic meatus was located on both axial (1a) and coronal (1b) planes. Subsequently, the hydrostatic column's vertical and sagittal dimensions were measured using the sagittal plane (2). This facilitated the application of the above-described formula to adjust for the hydrostatic pressure gradient between the sensor's position and the external acoustic meatus.
